# Supplementary material for: A computational lens into how music characterizes genre in film
Source: PLoS One. 2021 Apr 8;16(4):e0249957. doi: 10.1371/journal.pone.0249957 (PMC8031455; doi:10.1371/journal.pone.0249957)
Supplement: S1 Appendix — (PDF) [file pone.0249957.s001.pdf]

110-film corpus summary

Title	Genre Tags
300: Rise of an Empire	Action, Drama
Aladdin (2019)	Romance
Alita: Battle Angel	Action, Sci-Fi
Annabelle	Horror
Ant-Man	Action, Comedy, Sci-Fi
Ant-Man and the Wasp	Action, Comedy, Sci-Fi
Aquaman (2018)	Action, Sci-Fi
Avengers: Age of Ultron	Action, Sci-Fi
Avengers: Endgame	Action, Drama, Sci-Fi
Avengers: Infinity War	Action, Sci-Fi
Beauty and the Beast (2017)	Romance
Black Mass	Drama
Blended	Comedy, Romance
Bohemian Rhapsody	Drama
Captain America: Civil War	Action, Sci-Fi
Captain America: The Winter Soldier	Action, Sci-Fi
Captain Marvel	Action, Sci-Fi
Chappaquiddick	Drama
Christopher Robin	Comedy, Drama
Cinderella (2015)	Drama, Romance
Collateral Beauty	Drama, Romance
Crazy Rich Asians	Comedy, Drama, Romance
Creed	Drama
Doctor Strange	Action, Sci-Fi
Dora and the Lost City of Gold	Comedy
Dumb and Dumber To	Comedy
Dunkirk	Action, Drama
Edge of Tomorrow	Action, Sci-Fi
Entourage	Comedy, Drama
First Man	Drama
Focus (2015)	Comedy, Drama, Romance
Geostorm	Action, Sci-Fi
Going in Style	Comedy
Halloween (2018)	Horror
Happy Death Day	Horror
Hitman: Agent 47	Action
Horrible Bosses 2	Comedy
How to be Single	Comedy, Drama, Romance
In the Heart of the Sea	Action, Drama
Incredibles 2	Action, Comedy, Sci-Fi
Interstellar	Drama, Sci-Fi
Into the Woods (2014)	Comedy, Drama
It Chapter 2	Drama, Horror
Johnny English Strikes Again	Action, Comedy
Jumanji: Welcome to the Jungle	Action, Comedy
Jupiter Ascending	Action, Sci-Fi
Justice League	Action, Sci-Fi
King Arthur: Legend of the Sword	Action, Drama
Kingsman: The Secret Service	Action, Comedy
Kong: Skull Island	Action, Sci-Fi
Krampus	Comedy, Drama, Horror
Lights Out (2016)	Drama, Horror
Mad Max Fury Road	Action, Sci-Fi

Magic Mike XXL	Comedy, Drama
Maleficent	Action, Romance
Me Before You	Drama, Romance
Megan Leavey	Drama
Miss Peregrine's Home for Peculiar Children	Drama
Mission: Impossible - Fallout	Action
Mission: Impossible - Rogue Nation	Action
Moonlight	Drama
Mortal Engines	Action, Sci-Fi
Murder on the Orient Express	Drama
Need for Speed	Action
Oceans 8	Action, Comedy
Paddington 2	Comedy
Pan	Comedy
Parasite	Comedy, Drama
Pet Sematary	Horror
Pirates of the Caribbean: Dead Men Tell No Tales	Action
Pokemon Detective Pikachu	Action, Comedy, Sci-Fi
Queen of Katwe	Drama
Rambo: Last Blood	Action
Rampage	Action, Sci-Fi
Ready Player One	Action, Sci-Fi
Run All Night	Action, Drama
San Andreas	Action, Drama
Shazam!	Action, Comedy
Sicario	Action, Drama
Smallfoot	Comedy
Solo: A Star Wars Story	Action, Sci-Fi
Spotlight	Drama
Star Trek Beyond	Action, Sci-Fi
Star Wars: The Force Awakens	Action, Sci-Fi
Star Wars: The Last Jedi	Action, Sci-Fi
Stuber	Action, Comedy
Suicide Squad	Action, Sci-Fi
Sully	Drama
Tammy	Comedy, Romance
Teen Titans Go! To the Movies	Action, Comedy, Sci-Fi
The Boy	Horror
The Conjuring 2	Horror
The House with a Clock in Its Walls	Comedy, Horror, Sci-Fi
The Hustle (2019)	Comedy
The Imitation Game	Drama
The Intern	Comedy, Drama
The Judge	Drama
The Legend of Tarzan	Action, Drama, Romance
The Man from U.N.C.L.E.	Action, Comedy
The Meg	Action, Horror, Sci-Fi
The Peanut Butter Falcon	Comedy, Drama
The Post	Drama
This Is Where I Leave You	Comedy, Drama
Thor Ragnarok	Action, Comedy, Sci-Fi
Tolkien	Drama, Romance
Tomorrowland	Action, Sci-Fi
Transcendence	Drama, Sci-Fi
Vacation	Comedy
Venom	Action, Sci-Fi

Wonder Woman	Action, Sci-Fi
