# Supplementary material for: A computational lens into how music characterizes genre in film
Source: PLoS One. 2021 Apr 8;16(4):e0249957. doi: 10.1371/journal.pone.0249957 (PMC8031455; doi:10.1371/journal.pone.0249957)
Supplement: S2 Appendix — (PDF) [file pone.0249957.s002.pdf]

Precision-recall curves for top-performing MIR and VGGish models

MIR model (average pooling model, F1-score = 0.61)

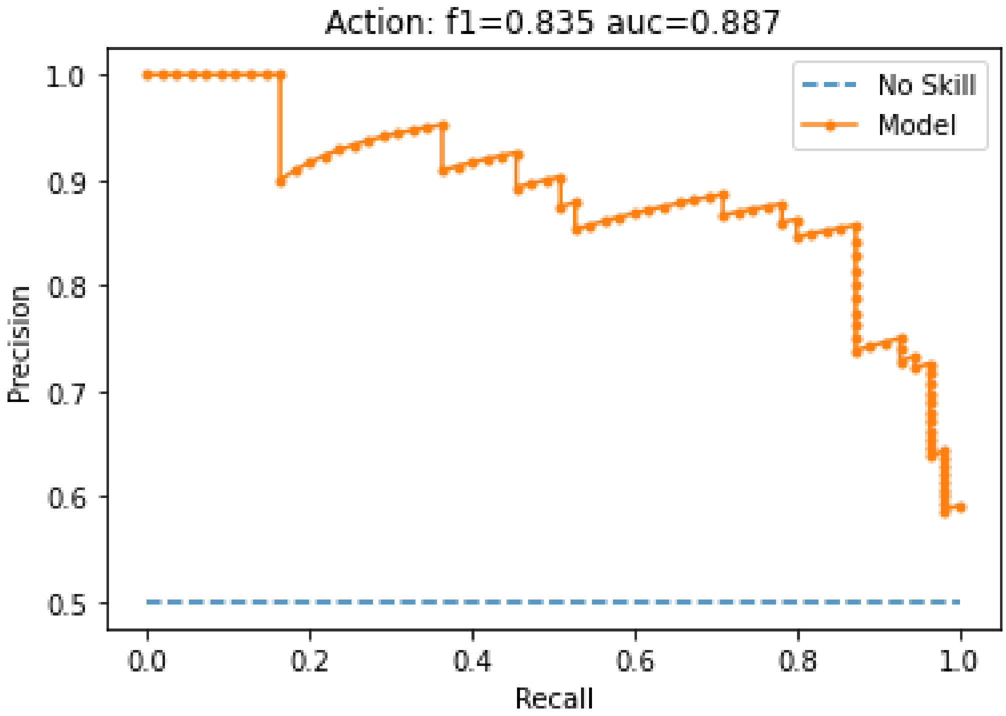

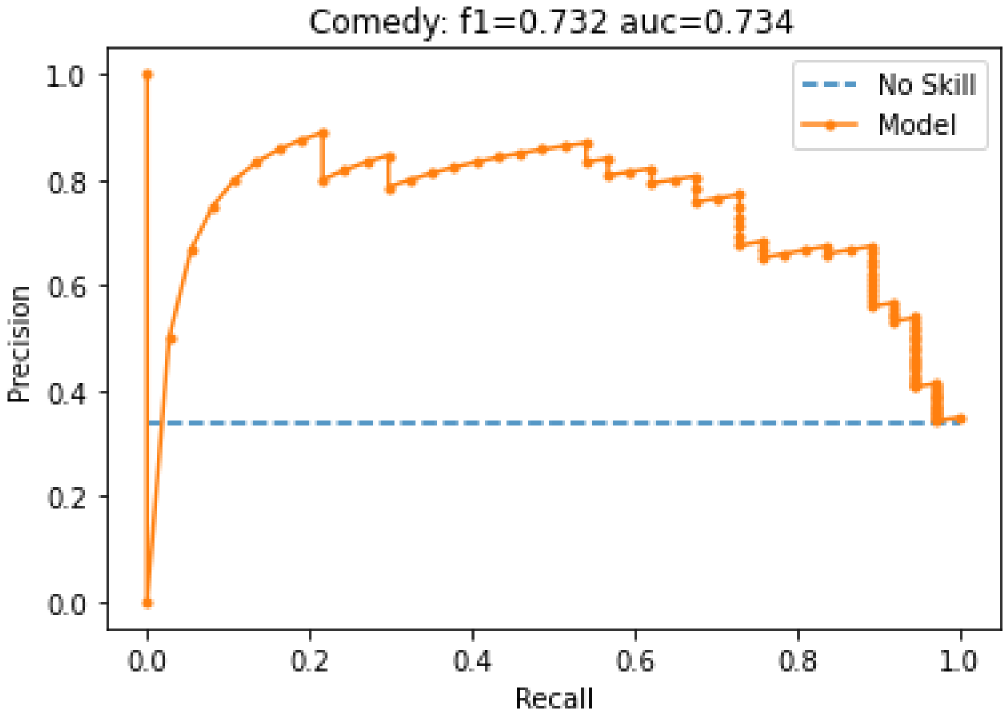

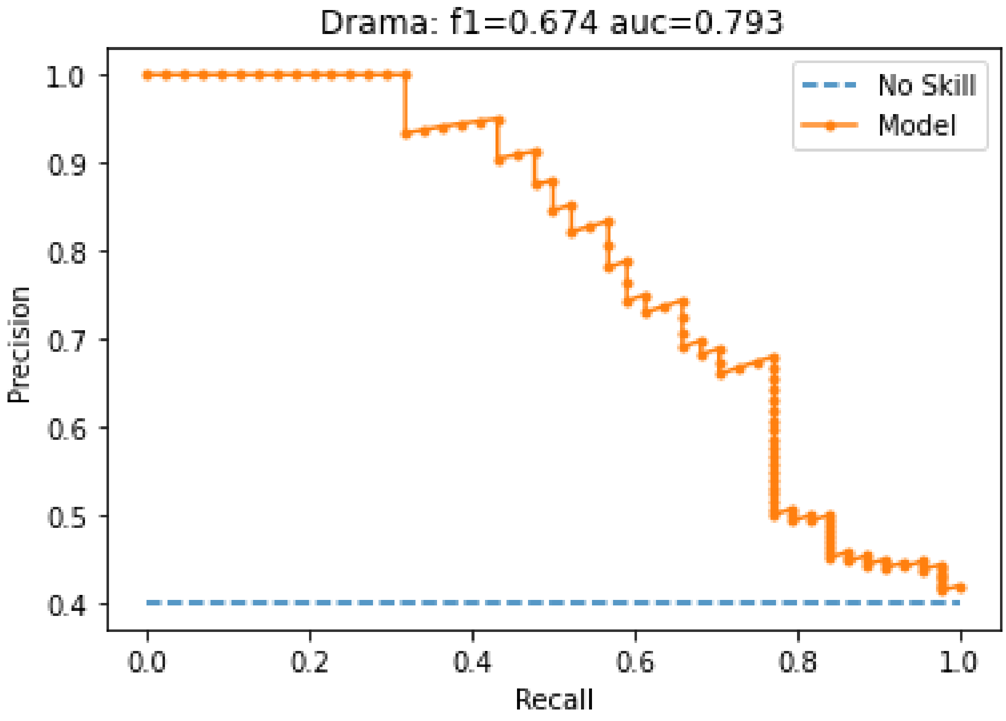
Drama: f1=0.674 auc=0.793
No Skill
Model
Precision
Recall
1.0
0.9
0.8
0.7
0.6
0.5
0.4
0.0
0.2
0.4
0.6
0.8
1.0

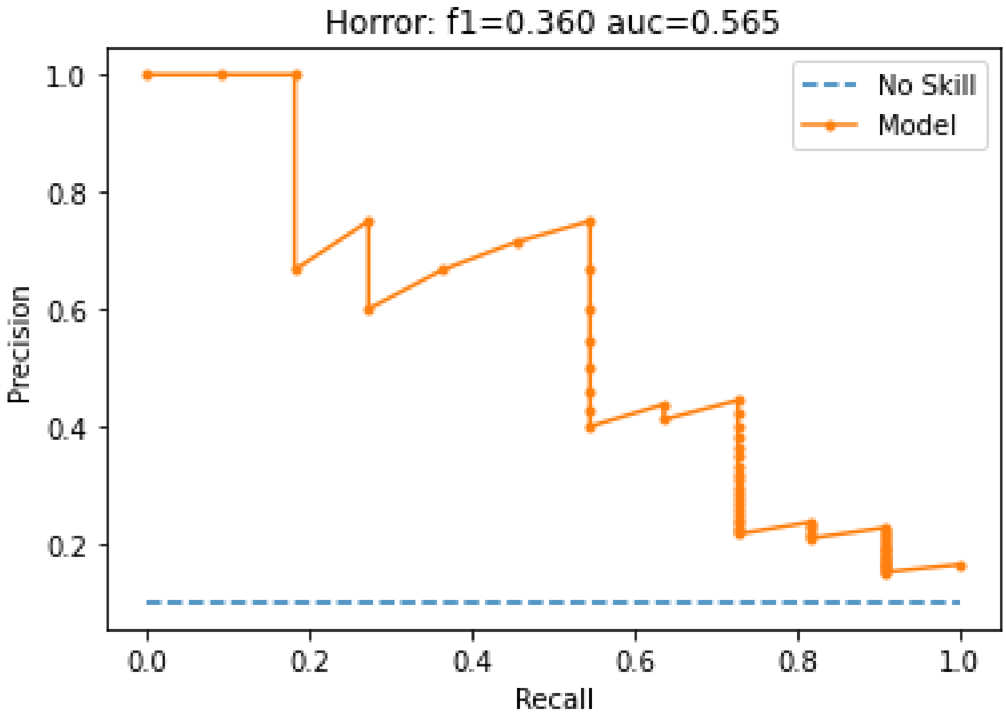
Horror: f1=0.360 auc=0.565
No Skill
Model
Precision
Recall
1.0
0.8
0.6
0.4
0.2
0.0
0.2
0.4
0.6
0.8
1.0

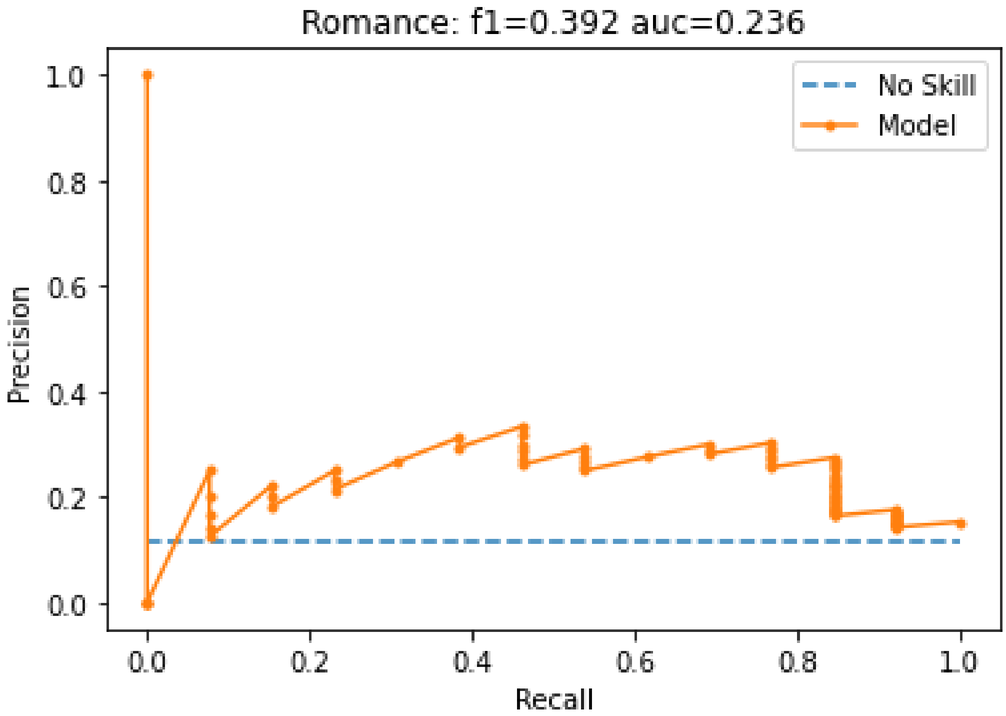
Romance: f1=0.392 auc=0.236
No Skill
Model
Precision
Recall
0.0
0.2
0.4
0.6
0.8
1.0

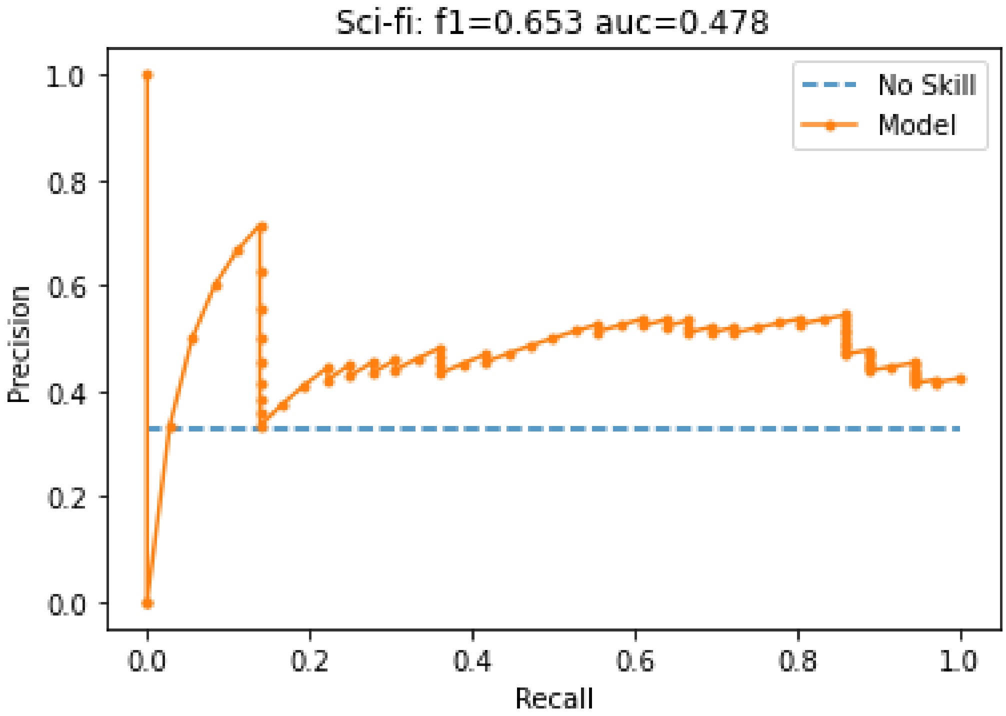
Sci-fi: f1=0.653 auc=0.478
No Skill
Model
Precision
Recall
0.0
0.2
0.4
0.6
0.8
1.0

VGGish model (single-attention pooling model, F1-score = 0.65)

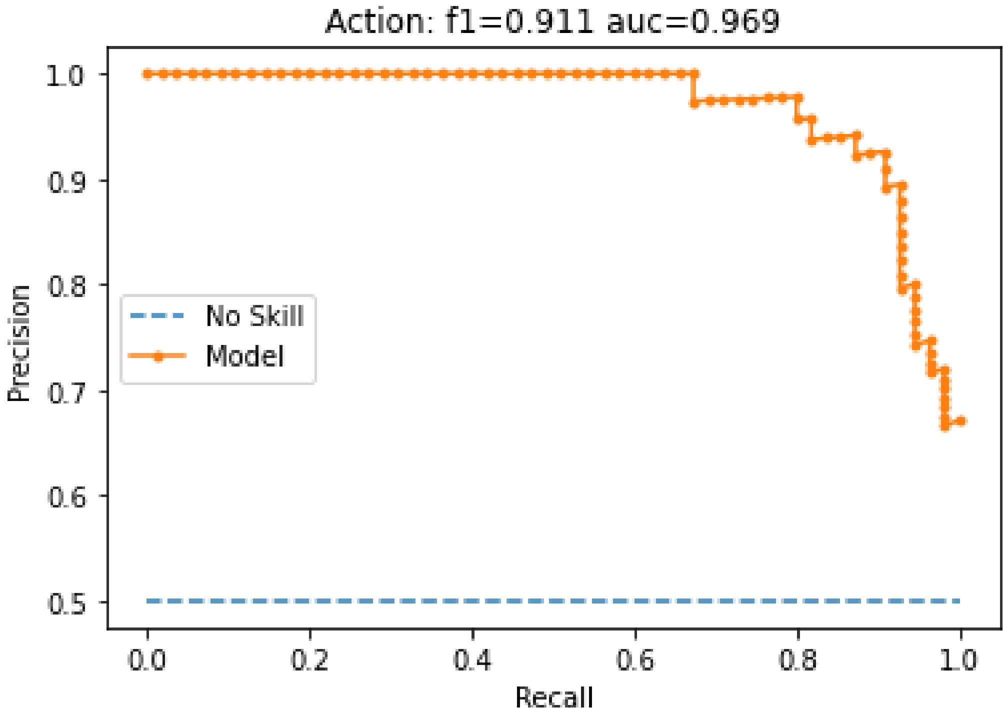

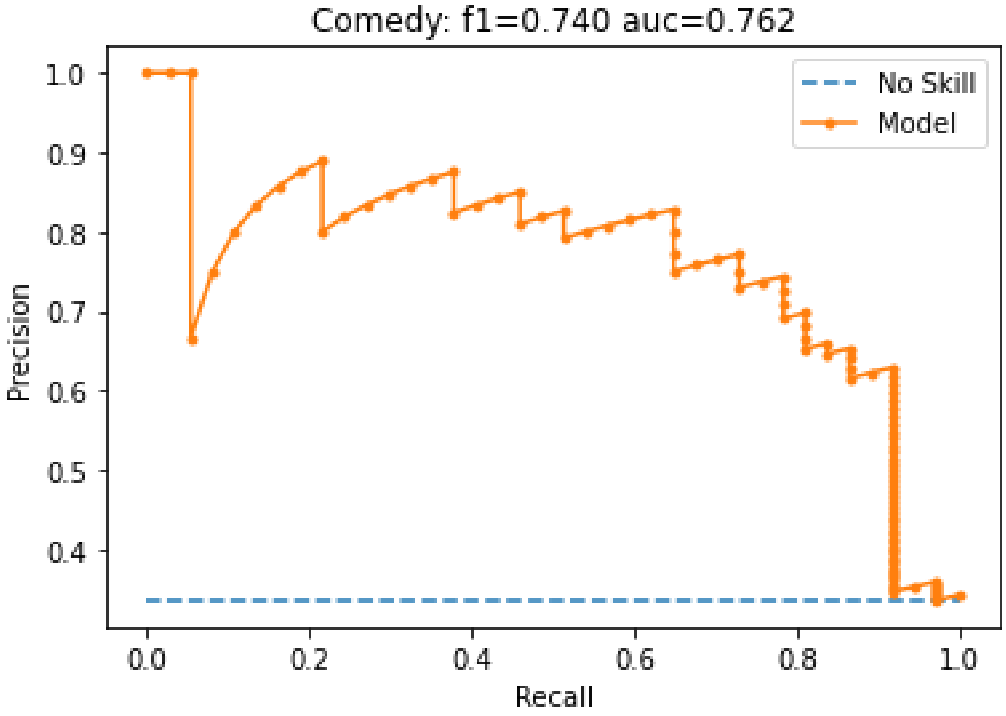

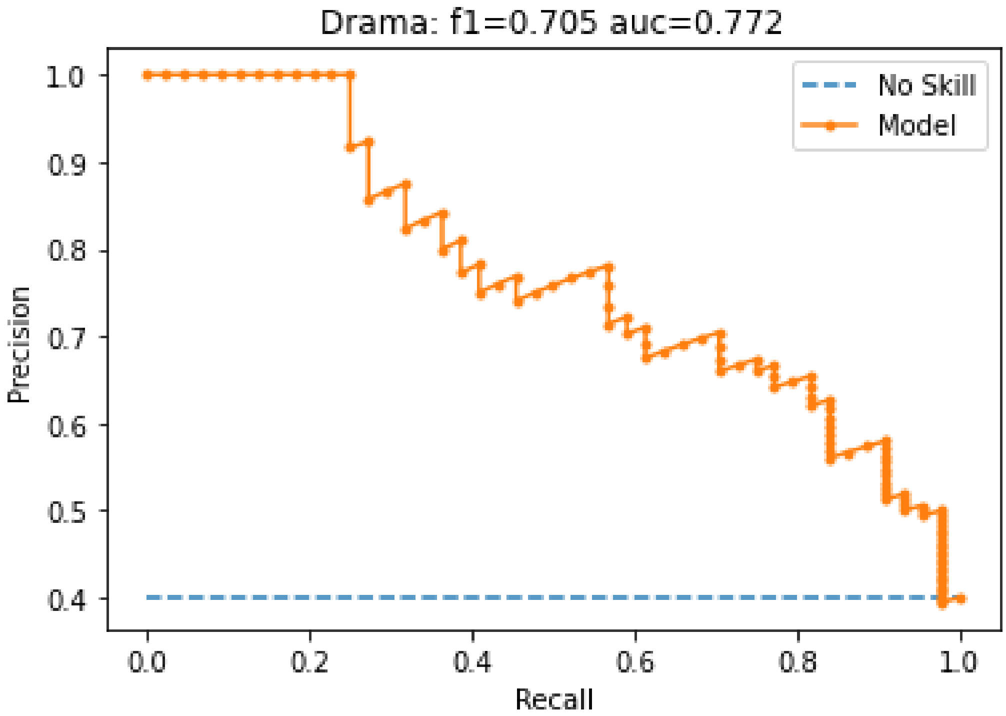
Drama: f1=0.705 auc=0.772
No Skill
Model
Precision
Recall
0.0
0.2
0.4
0.6
0.8
1.0
0.4
0.5
0.6
0.7
0.8
0.9
1.0

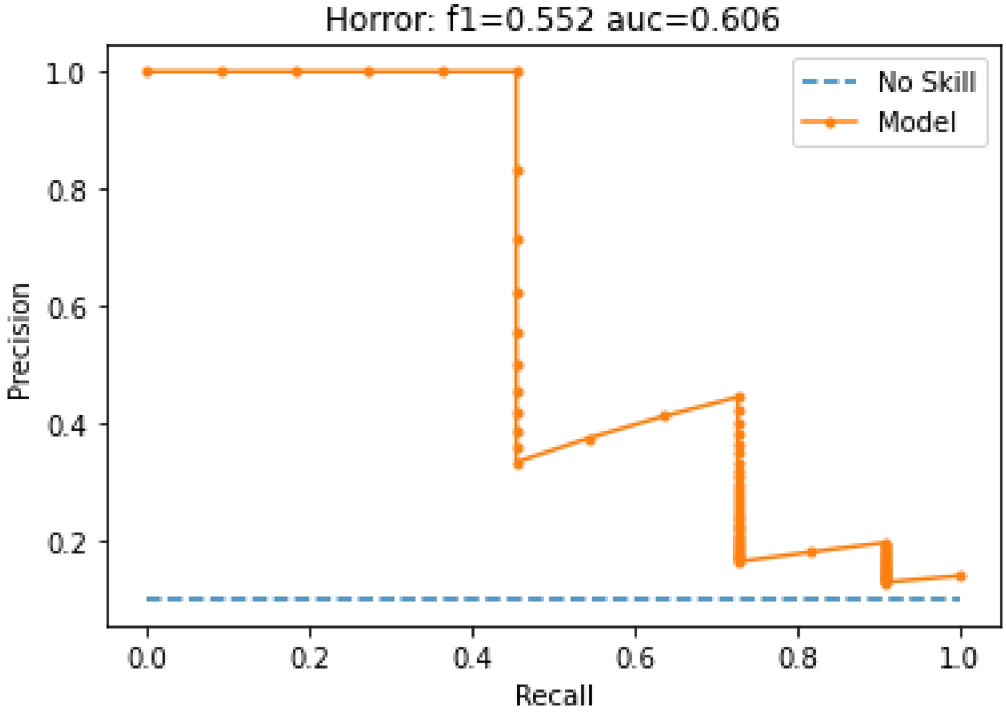
Horror: f1=0.552 auc=0.606
No Skill
Model
Precision
Recall
0.0
0.2
0.4
0.6
0.8
1.0
0.2
0.4
0.6
0.8
1.0

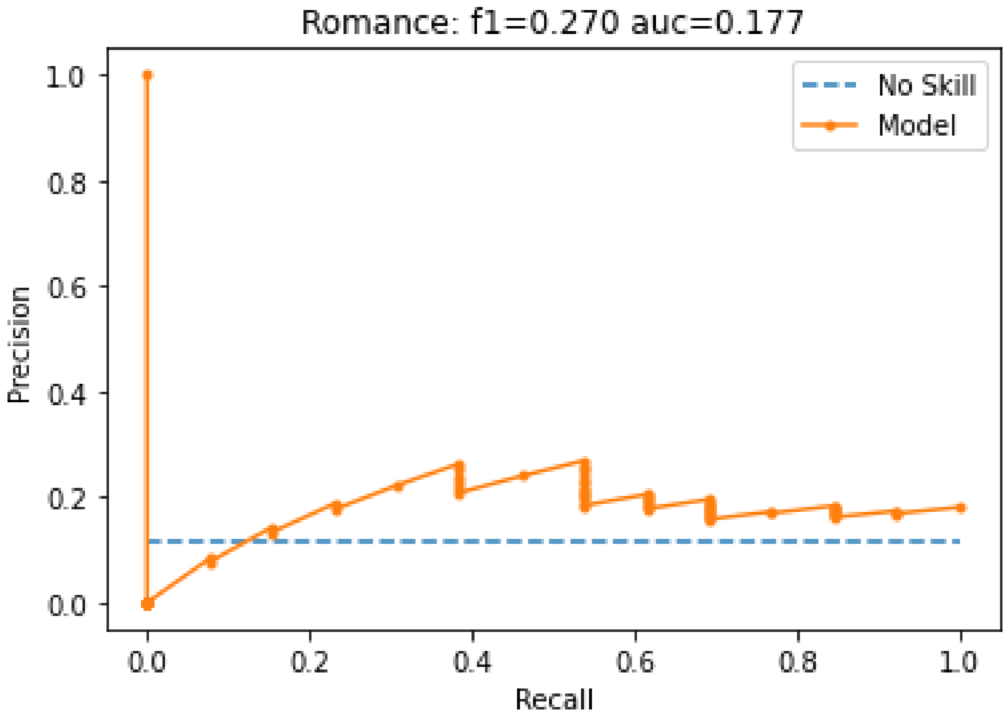
Romance: f1=0.270 auc=0.177
Precision
Recall
No Skill
Model
0.0
0.2
0.4
0.6
0.8
1.0

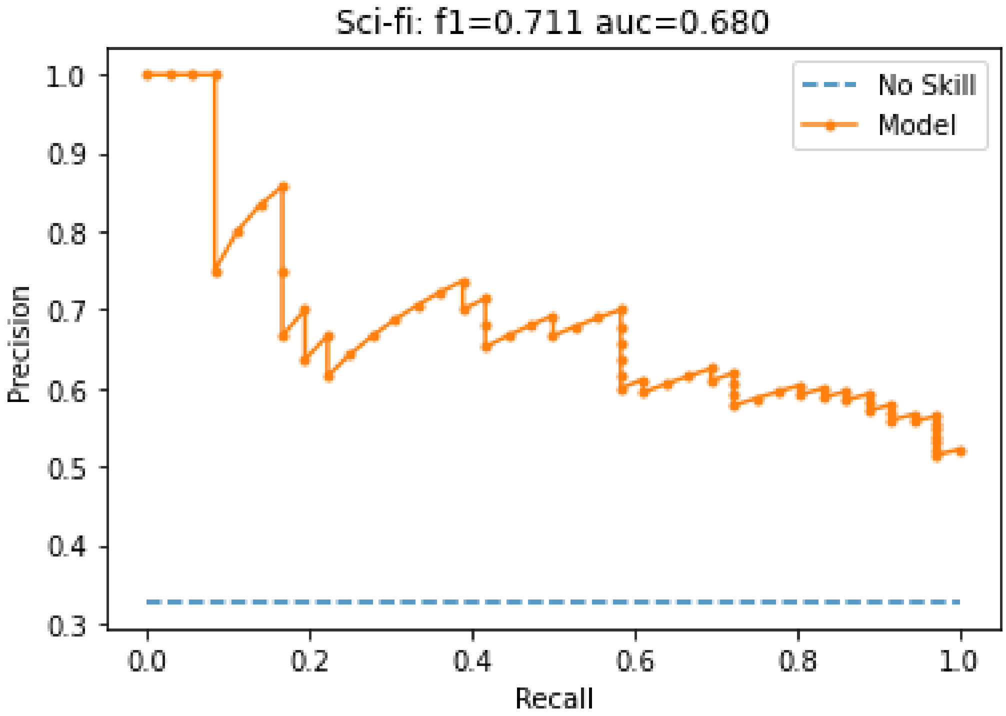
Sci-fi: f1=0.711 auc=0.680
Precision
Recall
No Skill
Model
0.3
0.4
0.5
0.6
0.7
0.8
0.9
1.0
0.0
0.2
0.4
0.6
0.8
1.0
