# Supplementary material for: A computational lens into how music characterizes genre in film
Source: PLoS One. 2021 Apr 8;16(4):e0249957. doi: 10.1371/journal.pone.0249957 (PMC8031455; doi:10.1371/journal.pone.0249957)
Supplement: S3 Appendix — (PDF) [file pone.0249957.s003.pdf]

Film-Level Precision-Recall Scatter Plot for VGGish and MIR Average Pooling Models

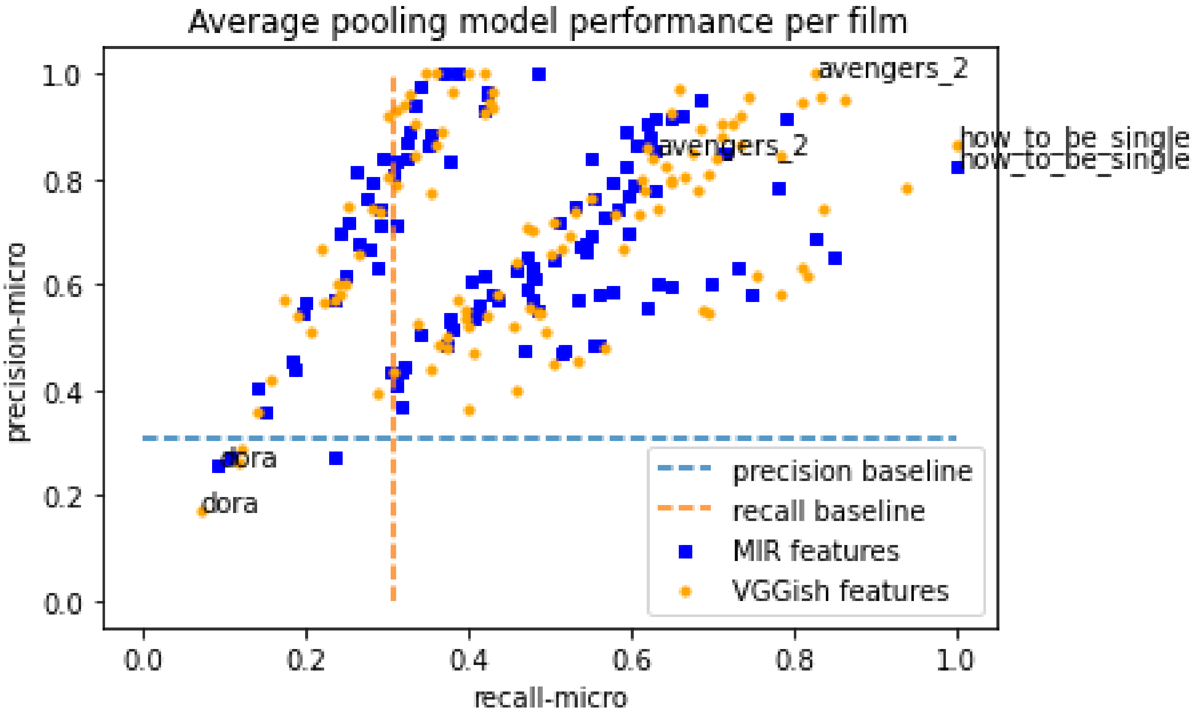

Scatter plot displaying precision and recall for each film (micro-averaged across all cues in that film), for both VGGish and MIR average pooling models. Average pooling models were chosen because they were the best-performing models that made independent predictions on individual cues. The films with the highest and lowest precision and recall values are labeled. The dotted lines display the precision and recall values of a random guess baseline classifier.
