# Supplementary material for: A computational lens into how music characterizes genre in film
Source: PLoS One. 2021 Apr 8;16(4):e0249957. doi: 10.1371/journal.pone.0249957 (PMC8031455; doi:10.1371/journal.pone.0249957)
Supplement: Attachment — Submitted filename: PLOS Rebuttal Letter FINAL.pdf [file pone.0249957.s005.pdf]

Benjamin Ma
University of Southern California
Los Angeles, CA, USA 90007
408-307-3768
benjamjm@usc.edu

"A computational lens into how music characterizes genre in film" (PONE-D-20-31042)
PLOS One

To the academic editor and reviewers:

Thank you for your insightful comments on our manuscript submission. We have reviewed your comments one-by-one and prepared a revised document with changes based on your feedback. In this letter, we describe each reviewer comment and the changes we have made to address it.

We received editorial feedback to amend our Competing Interests statement to acknowledge this study's partial funding from a corporation, Google. We have updated our Cover Letter to include an amended Competing Interests statement. Please let us know if other clarifications must be made to the manuscript in this regard.

We received feedback from reviewers advising us to provide more details about p-values and statistical significance of our results. To this end, we conducted Mann-Whitney U tests on our results from Table 5 and corrected them with Bonferroni correction.

To show that our results were replicable, we re-ran all of our experiments with leave-one-out cross-validation. We believe that this further strengthens the conclusions drawn from the experimental modeling results. At the behest of one of the reviewers, we also include scatter plots in the Precision-Recall space to better illustrate the flexibility and performance of our models.

We hope that the statistical tests that we have implemented in the revised submission will help further substantiate our experimental findings, and the conclusions drawn from our work.

One reviewer asked us to justify our choice of music information retrieval (MIR) features, as well as clarify how we calculated them. The MIR features we chose have shown utility in music emotion recognition and music genre classification from prior published work. Default parameters were used for computation and then texture-windowed to provide our models feature sets with equal window

lengths. In the manuscript, we have clarified how we computed our MIR features, and cited prior works which inspired our choice for the features used in our study. Our revised submission now includes specific details of which previous studies we were following and how we computed the features we used for our study.

We thank the reviewers for inquiring why the feature importance analysis was not conducted for VGGish features. We have added a sentence in our manuscript that mentions that VGGish features are not interpretable like MIR features are (for the reason that VGGish features do not correlate with human-understandable musical descriptors, such as loudness), so a feature importance plot for VGGish features would similarly not be interpretable. Our motivation for performing PFI was not to improve model performance, but rather to get a sense for which features contributed most to genre predictions. We have amended our manuscript to address this comment. Finally, we have added confidence interval indicators to Figure 3, which displays the results of the PFI analysis.

The reviewers advised us to more explicitly state the contributions and potential applications of our work. To address this feedback, we have added in a new section to our manuscript called "Contribution of this Work," which we hope will allow the reader to more precisely understand what we add to this area of study.

We appreciate the reviewers for pointing out a useful reference for cross-modal studies involving music. We have included the citation in our updated manuscript. Additionally, we have trimmed the "Visual-musical cross-modal analysis" section to avoid redundancy in the results section.

We received feedback that the "Multiple instance learning" sub-section in Related Works is unclear. In our most recent revision, we feel that the section is better motivated and clearer with regard to what a bag and an instance are in the context of our study. We believe the manuscript now better reflects why our problem could be framed as an MIL task, and we thank the reviewer for pointing out that this will allow the readers of our manuscript to better understand multiple instance learning and its relationship to this study.

A reviewer asked for more details on Score Stamper's performance on three films that were manually annotated in-house. We have added to our manuscript the number of instances per movie, as well as our justification for choosing the three movies (they are of different genres, by different directors, and contain different style soundtracks).

At the request of the reviewers, we have added more information about the models and architectures that we used in our study. Additionally, we have expanded our study to include leave-one-out error analysis, which we hope provides more rigor to our study, especially given the relatively small size of our dataset. In our new manuscript, we more clearly show our method's efficacy in predicting film genres and give more details about our models so that our study may be more easily reproduced. Finally, we have added a comparative analysis of our models' performance against the baseline classifiers and prior studies.

Thank you for the comments about the need for additional clarity in the conclusions on "Musical-visual cross-modal analysis". We have now added remarks on the various implications of our results. When we compare visual and musical styles by genre, the musical styles in question are the characteristics the model has learned to associate with every genre, which are not necessarily the actual musical characteristics that distinguish different film genres. Just as the model has learned an approximation of musical characteristics distinguishing film genres, our analysis approximates the relationship between visual and musical characteristics in different film genres.

Our goal in our musical-visual cross-modal analysis is to determine if visual features associated with a genre correlate with music that the model has learned to be characteristic of that same genre. We have updated the manuscript to clarify this motivation for readers.

We thank our reviewers for their interest in our manuscript and improving it. We hope that our work will find some use to our readers and others who are interested in understanding music's role as a significant and interactive cinematic device.
