# Supplementary material for: A computational lens into how music characterizes genre in film
Source: PLoS One. 2021 Apr 8;16(4):e0249957. doi: 10.1371/journal.pone.0249957 (PMC8031455; doi:10.1371/journal.pone.0249957)
Supplement: Attachment — Submitted filename: Rebuttal submission3 PLOSONE.pdf [file pone.0249957.s006.pdf]

Benjamin Ma
University of Southern California
Los Angeles, CA, USA 90007
408-307-3768
benjamjm@usc.edu

“A computational lens into how music characterizes genre in film” (PONE-D-20-31042)
PLOS One

We thank the reviewers for their feedback, which have helped us further strengthen our manuscript. Below we provide details of how we addressed their comments.

1) Statistical significance tests were only performed for the visual feature experiments and not for the VGG-ish and MIR features.

Response: We overlooked showing statistical significance tests for the experiments involving VGGish and MIR features in the previous manuscript. We have included a paired t-test on the mean average precision (mAP) of our models, where the null hypothesis is that the mAP of our proposed model is not shown to be significantly higher than the random guess baseline. Perhaps not surprisingly, our best-performing models show significantly better performance than the baseline at the 0.01 level. The table has been updated with more details, and we hope the readers will see that these models are significantly better-performing than our baselines.

2) The caption of table 5 is unclear about what the bold style means: authors say to have computed p-values using bonferroni correction; this means that they have compared multiple tests, but I cannot understand what are these tests: did they compare all the tests for which they show the average at once? or did they compared "actual", "predicted" and "false positives" in each row? or all rows of "predicted" and then all rows of "actual" and then all rows of "false positives"?

Response: We appreciate the feedback about our caption in table 5: we have clarified this caption to indicate that we were using 6 hypotheses in our Bonferroni Correction, which correspond to comparing the median of a particular genre in a particular column (“Actual,” “Predicted,” and “False Positives”) with the median of *all of the other genres in that same column*. Our caption has been reworded and proper parameters are included to elucidate the tests that we ran.

3) Moreover, statistical tests need the same cardinality between the set tested. This would mean that the number of "films labeled with a given genre" is the same as the number of "films excluding the given genre", which seems unlikely. How they managed this problem for the statistical test?

Response: While there are some statistical tests that need the same cardinality between the two samples tested, we used a Mann Whitney U test in our results reported in Table 5, which does not mandate that the number of films labeled with a given genre is the same as the number of films excluding the given genre.

4) I appreciate the addition of supplementary figure S2, but it shows precision-recall curves, not scatter plots. PR-curves comes out when the classification is made based on some threshold, and they are a method for evaluating the model, not the distributions of the predictions. AUC (Area under curve) is similar to F1-score and in this case PR-curves don't add any knowledge. Even if other reviewers find this plot useful, it's not clear what "no skill" line is. It would be more clear if MIR and VGG-ish points were on the same plot. When I wrote "scatter plot in a precision-recall space", I was meaning a scatter plot, not a curve. Scatter plots are plots which shows points in their coordinates; in my example, coordinates were precision and recall. Points could be, for instance, each film. Multiple distributions can be plotted using different colors, eg. multiple models: see for instance the following image https://www.researchgate.net/profile/Mohit_Bansal7/publication/267783907/figure/fig3/AS:669377024258060@1536603327607/BCubed-Precision-Recall-scatter-plot-for-the-Japanese-English-dataset-Each-point.png A plot such that could be useful to qualitatively evaluate the difference models without falling in type I and type II errors.

Response: We appreciate our reviewer's clarification about the PR plot. We have amended our manuscript to include models based on VGGish features and MIR features on the same PR plot to facilitate easy comparison of models, as was shown in the reviewer's example. Each film's cues were compiled and the predictions of these cues were used to calculate micro-averaged precision and recall scores for a film. We label the highest- and the lowest-precision and recall films, which we believe indicates that the performance between models using MIR features and VGGish features is similar for each film. We hope this new figure complements the PR curves shown in supplementary figure S2 by allowing readers to easily compare between MIR and VGGish-based models.
